# FACTORS ASSOCIATED WITH HELP-SEEKING AMONG US CHINESE OLDER ADULTS WHO REPORTED ELDER MISTREATMENT

**DOI:** 10.1093/geroni/igad104.2103

**Published:** 2023-12-21

**Authors:** Ying-Yu Chao, Jin Young Seo, Yu-Ping Chang

**Affiliations:** Rutgers, the State University of New Jersey, Somerset, New Jersey, United States; Hunter College, CUNY, New York City, New York, United States; University at Buffalo, The State University of New York, Buffalo, New York, United States

## Abstract

**Purpose:**

Elder mistreatment (EM) is an important public health issue. Guided by the Andersen’s Behavioral Model of Health Services Use, this study aimed to examine factors that impede or facilitate help-seeking intentions and behaviors among U.S. Chinese older adults who reported EM.

**Methods:**

Data were from the Population Study of Chinese Elderly in Chicago (PINE). Different types of mistreatments, informal and formal help-seeking intentions and behaviors were assessed. Descriptive statistics, linear regression, and multinominal logistic regression were used.

**Results:**

Among 450 participants who reported EM, 46.55% experienced psychological mistreatment, 29.91% had financial exploitation, 14.92% had caregiver neglect, 4.68% had physical mistreatment, 1.11% had sexual mistreatment, and 17.78% had multiple victimizations. Intentions of informal help-seeking was higher among victims who were female and married, and those who experienced financial exploitation and caregiver neglect. Formal help-seeking intentions were lower among victims who had a larger social network size. EM victims who were female, married, perceived more social support, experienced financial exploitation, and experienced caregiver neglect were more likely to seek help from informal resources. EM victims who had higher income, perceived more social support, and had multiple victimizations were more likely to seek formal resources. Conclusion/implication: Our findings suggest that the predisposing factors, enabling resources, and need factors that impacted seeking help from informal and formal resources among older Chinese Americans who reported mistreatment. Findings provided important insights for developing early screening programs and interventions, such as increasing help-seeking channels and improving access to care services.

